# The Physical Signs of Lobar Pneumonia

**Published:** 1904-03-19

**Authors:** 


### Hospital Clinics and Medical Progress.

### THE PHYSICAL SIGNS OF LOBAR PNEUMONIA.

The importance of an early recognition of lobar pneumonia by means of definite physical signs is emphasised by the acute course of the disease. This is especially the case in those forms of pneumonia that sometimes closely simulate the symptoms of grave intra-abdominal lesions. The physical signs of the stage of consolidation are fairly definite and easily recognised, but the phenomena presented by the initial stages often vary within wide limits. This variation has led to a divergence of opinion as to the most characteristic feature in the physical signs present at the beginning of a lobar pneumonia. The signs to which most importance has been given in this stage are an impaired percussion resonance over the affected area and the presence of the crepitant râle. This later sign was first pointed out by Laennec, who was strongly in favour of its diagnostic value. Other observers say that either they have not found it pathognomonic of lobar pneumonia, or else they have but rarely heard it.

Another sign that has been observed is an alteration in the character of the breath-sounds. Stokes insisted that the breath-sounds are of a more "puerile" character in the affected part. Grisolle, however, never observed this harshness of breathing as the first auscultatory sign, and held that a diminution of the normal respiratory murmur with a loss of purity of the breath-sounds was one of the first phenomena. This view was supported by Fuller. Walshe's opinion is that the sounds heard over the affected area are weaker, harsher, and of a higher pitch. He also has pointed out that sounds indicative of exaggerated breathing may be heard when the lesion is deep-seated. Wilson Fox states that an increased harshness of the murmur may be the first phenomenon noticed, but this is not always the case. Sometimes the respiratory murmur is weakened, closely resembling the "indeterminate" breathing of Skoda.

Juergenson, however, maintains the importance of the crepitant râle as the first among the auscultatory signs of pneumonia. The breath sounds are also modified, sometimes puerile and harsh, sometimes weak but still vesicular. Osler has also noted a frequent diminution in the vesicular murmur during the first stage of lobar pneumonia. Strumpell, however, makes no mention of any characteristic alteration in the breath-sounds. Aufrecht, indeed, says that the crepitant râle remains the characteristic symptom of the inflammatory invasion of the lung, though there is also, in many cases, a prolongation of the expiratory murmur.

From the divergence of the above opinions it is seen that no definite conclusion as to the characteristic physical signs of early lobar pneumonia has yet been reached. The different views may be classified as follows: (1) No change in the breath-sounds, the crepitant râle the only characteristic sign of pneumonia; (2) the breath-sounds usually abnormal, being harsh or puerile in character; (3) a diminution of the respiratory murmur the chief sign; (4) no constant change in the auscultatory signs during the initial stages of pneumonia.

Recently Conner and Dodge (*Amer. Jour. Med. Sci.*, 1903) have recorded a number of observations in a series of cases of lobar pneumonia. These cases were examined with a view to ascertain the relative importance of the different physical signs in the early stages of the disease.

In the first place they draw attention to two points in the examination of the patient: (1) The patient must be made to breathe through the nose. This is because when breathing through the mouth the expiratory murmur becomes tubular in character, masking the true vesicular sounds. (2) The patient should be examined in the sitting position whenever possible. When lying on the side the percussion note of the lower side is apparently more resonant, and the resistance offered to the examining finger is also different on the two sides. The auscultatory sounds also show a distinct difference in character, being more tubular on the lower compressed side. These difficulties are avoided by examining the patient in the sitting position.

Taking the average result of their series of observations, Conner and Dodge find that in 79 per cent. of their cases the respiratory murmur was diminished in the early stages of the disease. The area over which the breath-sounds were enfeebled corresponded fairly closely with the area of impaired percussion resonance. Over this area the respiratory murmur was not only feeble, but indistinct in quality. This change progressed with the illness, the diminution of the vesicular murmur proceeding until, just before the appearance of tubular breathing, the breath-sounds were almost suppressed. Indeed, this absence of breath sounds and impaired resonance gave a close resemblance to the physical signs of pleuritic effusion.

The enfeeblement of the respiratory murmur appears early in the illness, usually within 24 hours of the onset of the disease. At the same time there is usually distinct impairment of percussion resonance over the same region, but sometimes the change in the breath-sounds exists before any diminished resonance can be detected. In a few cases the converse happens—*i.e.* impaired resonance is noticed before any change in the breath-sounds.

The first stage lasts for one or two days before any definite bronchial breathing can be heard. This stage of diminished and enfeebled breath sounds apparently corresponds with the stage of engorgement of the pulmonary capillaries and œdema of the mucous membrane. A corresponding condition exists in the lungs in uncompensated cardiac lesions. This connection between the physical signs and pathological lesion has been confirmed by the experiments of Hartwell, of New York.

In addition to the changes in the vesicular murmur, crepitant râles and friction-sounds were heard in 50 per cent. of the cases recorded by Conner and Dodge, being present before the advent of true bronchial breathing. In 35 per cent. more râles of some kind were heard in the initial stage.

The results of the observations of Conner and Dodge would place importance on the following physical signs in the early stage in this order: (1) A diminution of the normal vesicular murmur—

*i.e.* indistinct breath-sounds, when the patient is in the sitting position; (2) Impaired percussion resonance over the affected area; (3) The presence of the crepitant râle; (4) Increased vocal resonance.

In the second stage of pneumonia the signs are usually definite and distinct. Bronchial breathing and bronchophony appear with complete hepatisation of the lung tissue. The bronchial quality of the breathing is first noticeable during expiration. When first present it is soft and low-pitched. This period of broncho-vesicular breathing is usually of brief duration. Both inspiratory and expiratory sounds grow louder and harsher, until they become the intense breath-sounds associated with consolidation—the harsh and intense inspiratory and expiratory murmurs of bronchial breathing. These signs appear in small areas at first, and gradually extend over the affected area.

The percussion signs in the second stage are a marked dulness over the lesion, with a distinct increase of resonance in the healthy lung overlying the consolidated portion. For example, if the posterior part of the base of the right lung is affected the middle lobe anteriorly is hyper-resonant. This resonance may even resemble that of Skoda.

Bronchophony usually corresponds with the bronchial breathing in its appearance and disappearance.

The physical signs of resolution usually occur in the inverse order to those of onset. The bronchial breathing becomes lower-pitched and fainter, the bronchial expiratory murmur disappearing last. There is, however, one exception noticed by Conner and Dodge. The low-pitched bronchial breathing does not merge into the feeble breath-sounds of the early stage, but is followed by a harsh vesicular murmur.

Resolution takes place in a variety of ways. In some cases the physical signs alter before defervescence, but when the temperature does fall it is by crisis. This class of case gave the best example of redux crepitation. In by far the greater number, however, resolution coincides with the fall of body temperature, and in these the temperature declined by lysis. Others there are in which, though defervescence occurs at the usual time, complete consolidation persists for some days, without any other complication. In these there is an almost complete absence of redux crepitations.

